# Effect of Instructions Emphasizing Velocity or Accuracy Given in a Random or Blocked Order on Performance Testing and Kinematics in Dart Throwing

**DOI:** 10.3389/fpsyg.2019.01359

**Published:** 2019-06-12

**Authors:** Roland van den Tillaar, Tore Kristian Aune

**Affiliations:** Department of Sports Science and Physical Education, Nord University, Bodø, Norway

**Keywords:** overarm throwing, coordination, velocity, accuracy, Fitts’ law

## Abstract

The aims of this study were to investigate the effects of throwing instruction (i.e., velocity and accuracy) and testing order (random or blocked) on dart throwing performance testing and on the movement strategies leading to this performance. Twelve physical education students (nine men and three women, age: 24 ± 7.5; mass: 77.7 ± 15.8, height: 1.77 ± 0.06 m) performed dart throws with four different instructions, varying in priority regarding velocity and accuracy, instructed in a blocked and random manner. The main findings were that dart velocity decreased when the priority of accuracy increased. However, when accuracy was the main priority, accuracy increased only when measured for consistency. Testing order influenced peak joint kinematics of wrist flexion in addition to finger extension and the time of occurrence of elbow extension. Instructions emphasizing velocity and/or accuracy showed a clear speed-accuracy trade-off in dart throwing and thereby followed Fitts’ law. Testing order had a minor effect on the speed-accuracy trade-off. The blocked testing order appeared to increase performance outcomes that favored the priority set by the instruction in contrast to the random test order. These differences were based upon adjustments of joint movements, which were based upon the knowledge of the previous attempts. These adjustments were visible between the different instructions through changes in the execution timing of peak wrist flexion and elbow and finger extension.

## Introduction

Throwing performance is an important skill in many sports. It consists of two main priorities, the velocity and accuracy of the throws. In some sports like javelin, velocity is one of the most important factors, while the velocity and accuracy of throwing are equally important in many other sports like such as baseball, handball, and cricket (van den Tillaar and Ettema, 2003a,b, 2006). There are also sports like darts in which the outcome is primarily based on the accuracy of the throw. Indermill and Husak (1984) found that the goal of the task influenced throwing accuracy and velocity in an inverse manner. This was indicated by using different types of instruction, i.e., “throw hard (100%), medium (75%) or soft (50%)” (Indermill and Husak, 1984). They showed that throwing accuracy was the highest when throwing with medium velocity, while throwing accuracy decreased when instructed to throw soft or hard. On the other hand, van den Tillaar and Ettema (van den Tillaar and Ettema, 2003a,b, 2006) showed that the type of instruction prioritizing velocity, accuracy or both (five instructions varying from throw as hard as possible to hit the target) influenced the velocity of overarm throwing in handball, but not the accuracy. The authors explained that the characteristics of the task govern the lack of the appearance of the speed-accuracy trade-off in overarm throwing in team handball. In handball, the main priority of the throws is velocity that will allow the ball to surpass the goalkeeper. Etnyre (1998) showed that in dart throwing, where accuracy was the main priority, accuracy decreased when emphasizing velocity. This is an indication that the type of task in the sport could be of importance for the velocity-accuracy trade off (Fitts, 1954). Etnyre (1998) only used two instructions: “throw normally” or “throw as hard as you can,” which makes it difficult to compare the study by Etnyre with studies in which several instructions were given, with different degrees of priority for the velocity and/or accuracy of throwing (van den Tillaar and Ettema, 2003a,b, 2006). Furthermore, Etnyre (1998) did not conduct kinematic analysis, which would give additional information about possible changes in throwing strategies (magnitude and timing of the different joint movements).

In addition, the way these throwing performances were tested could influence outcomes. Thus, van den Tillaar and colleagues (van den Tillaar and Ettema, 2003a,b, 2006) tested the effect of instruction on throwing performance in a random order, while Etnyre (1998) and Indermill and Husak (1984) tested instructions in a blocked order. This different organization of testing schedules could show variations that influence performance of a movement task (Hall and Magill, 1995; Pedersen and Loras, 2017). There is controversy on how a blocked versus a randomly organized testing schedule influences motor performance, and whether the best test results are provided by a blocked or randomly organized order of testing conditions (Magill and Anderson, 2007). Blocked testing implies that the same skill or condition is performed for a given period or number of trials before the next variation is introduced (Goode and Magill, 1986). In contrast, random testing requires that the different skills or conditions are presented in a random order (Rose and Christina, 2006). In learning new tasks, this phenomenon is called contextual interference (Shea and Morgan, 1979). Early studies of high contextual interference (random) showed an increased retention and transfer of motor skills, especially under changed contextual conditions (Shea and Morgan, 1979), and is also consistent with learning studies of cognitive tasks with high contextual interference (Battig, 1979). It is suggested that blocked testing and practice (low contextual interference) enhance performance accuracy in motor tasks where the same response structure is required for all trials (Lee and Magill, 1983, 1985; Li and Wright, 2000), while others have demonstrated that random is superior compared to blocked group testing and practice (Immink and Wright, 1998; Li and Wright, 2000). Under random testing, performers learn to produce movement patterns as accurately and quickly as possible, but also how to attune and switch flexibly from one trial to the next. A way to introduce contextual interference might be to introduce verbal instructions. Verbal instructions can be described as intrinsic contextual interference, and a random use of different types of verbal instructions probably create variable processing strategies for the subjects. Such increased contextual interference under practice might induce the subjects processing of task strategies to be learned, and thus facilitate performance between trials.

It is explained as that the deeper processing or reconstruction of the action plan during learning phase increases performance accuracy of random condition on tests (Lee and Magill, 1983, 1985; Shea and Titzer, 1993). However, Russell and Newell (2007) showed that high contextual interference (random) during training was not superior compared to low contextual interference (blocked). It was concluded that there are no general learning advantages or more persistent learning by using random versus blocked training, as broadly as those promoted in earlier literature. However, all these studies investigated a learning effect from practice, while the acute effect of blocked or random order during testing is not investigated much. It has been proposed that participants would structure the repeated sequences differently under blocked testing conditions compared to random testing conditions (Povel and Collard, 1982). If this were the case, differences in performance accuracy (outcome) and kinematics would arise in the respective conditions. Under blocked testing, one might predict that participants would attempt to restructure the sequences based upon the previous movement trial, and thereby, attune their movements to increase the outcome asked by the instruction (velocity and/or accuracy). In random testing, knowledge of result from the previous trial cannot be used for the next trial and thereby the subject misses the opportunity to attune the movement get a performance improvement. This contextual interference (Shea and Morgan, 1979) is widely investigated in motor learning, but to the best of our knowledge not much is known about the acute effects of ordering conditions (blocked or random) upon performance during testing, which is of main importance when evaluating motor performances.

Therefore, the aim of this study was to investigate the effects of throwing instruction on dart throwing performance testing (i.e., velocity and accuracy) and testing order (random or blocked) on movement strategies (kinematics) leading to this performance. It was hypothesized that performance outcome would follow Fitts’ law (Fitts, 1954), which suggests that if the velocity was the main priority, the throwing velocity would increase and the accuracy would decrease, while the velocity would decrease if accuracy was the main priority. In addition, it was predicted that blocked organization testing would be beneficial for performance accuracy and throwing velocity because participants could structure the repeated movement sequence and adjust efficiently from one trial to the next.

## Materials and Methods

### Participants

Twelve physical education students (nine men and three women) participated in this study. All subjects had some experience in dart throwing at a recreational level only, not at a competitive level (age: 24 ± 7.5 years, mass: 77.7 ± 15.8 kg, height: 1.77 ± 0.06 m). Before participating in this study, the subjects were fully informed about the protocol and informed written consent was obtained prior to all testing from each subject with the approval by the Norwegian Centre for Research Data (NSD) and a further approval by an ethics committee was not required as per applicable institutional and national guidelines and regulations.

### Procedure

After a general warm-up of 5 min, which included throwing drills to warm up the throwing arm (around 8–12 practice throws), the dart-throwing performance with a regular dart was tested from 2.37 m from a standard dartboard. The center of a dartboard (bulls) was hanging at a height of 1.73 m. Four different instructions were prescribed to the subjects, which were clarified beforehand, so that the subjects understood the exact meaning of the different instructions. These instructions, described previously in handball throwing and soccer kicking (van den Tillaar and Ettema, 2003a,b, 2006; van den Tillaar and Ulvik, 2014; van den Tillaar and Fuglstad, 2016), are summarized below.

For the first instruction, throwing as fast as possible was the only priority, with no concern for accuracy. This condition was used to determine the maximal dart velocity (*V*_0_). For the second instruction, the main priority was to throw as fast as possible and the secondary priority was to throw accurately (*V*_A_). For the third instruction, the opposite was instructed. The main priority was accuracy and the second priority was velocity (*A*_V_). For the fourth instruction, the only priority was to hit the target (*A*_0_) with no constraints set on velocity. The verbal instructions were given as follows:

(*V*_0_) Throw the dart *as fast as possible*, straight forward at the *dartboard.*

(*V*_A_) Throw the dart *as fast as possible* and *try to hit* the *bull.*

(*A*_V_) *Hit* the *bull* and *try* to throw *as fast as possible*.

(*A*_0_) *Hit* the *bull.*

Each subject was instructed to throw 10 times according to each instruction condition, resulting in 40 throws per subject. On the first day, the different instructions were given in a random order to avoid learning, fatigue, or any other practice related effect that might affect the results in a systematic way. The random order was based on a random number generator. The subjects had approximately 30–45 s rest between each throw. On the second day, the instructions were given in a blocked manner of 10 throws with one specific instruction before the next instruction. The order of the instructions in blocks was randomized for each subject to avoid a specific influence of order of the blocks that might influence the performance in a systematic way. There was at least 2 weeks between the two testing days in which no practice of dart throwing was allowed, to avoid any learning effect from test day one and two. This was also shown by some earlier studies (Lee and Simon, 2004; Maslovat et al., 2004; Russell and Newell, 2007) that found a minimal or nonexistent learning effect of this amount of random testing to the next session over such a period.

### Measurements

The throwing velocity of the dart was calculated from recordings from a 3-D digital video movement analysis system of six cameras positioned in a half-circle around the throwing position (QTM, sample rate 500 Hz). Reflective markers (2.6 cm diameter) were used to identify anatomical landmarks: (1) shoulder: acromion process on the side of the throwing arm, (2) elbow: lateral epicondyle of the throwing arm, (3) wrist: ulnar and radial styloid process of the throwing arm, (4) proximal and distal inter phalanges of phalanges III, and (5) dart: on top of the dart. Linear velocity of the dart was calculated using the absolute displacement in 3-D and a differential five-point filter. The time of release was derived from the change in length between the marker and the marker on the dart. The moment the dart left the hand, the distance between the distal inter phalanges marker and the dart marker increased abruptly and dramatically (van den Tillaar and Ettema, 2003b, 2007, 2009a,b). The joint angular velocities of the elbow extension, wrist flexion, and finger extension were derived from relative positions of the distal segment relative to the proximal segment of the joint of interest (Feltner and Dapena, 1989; Fradet et al., 2004; van den Tillaar and Ettema, 2007). All calculations were performed in Matlab 6.1 (The Mathworks Inc., Natick, MA, USA). Timing of peak angular velocity of the elbow, wrist, and finger joint movements were presented as the time before dart release.

Throwing accuracy was measured with a video camera (Panasonic model WV-F350E) 6 m from the dartboard. The field of vision between the camera and the dartboard was visible the entire time. The position of the dart was measured at the moment the dart struck the dartboard. Mean radial error, bivariate variable error, and centroid error as described by Hancock et al. (1995) and van den Tillaar and Ettema (2003a) were used as measurements of accuracy. Mean radial error (MRE) was measured as the average of absolute distance to the bull. Each subject’s midpoint was measured as the average hit location over all trials for each instruction. The absolute distance of a subject’s midpoint to the bull is called the bias or centroid error (CE). The bivariate variable error (BVE), also referred to as consistency, was measured as the average of the absolute distance to the subject’s own midpoint.

### Statistics

Levene’s test was used to check for homogeneity of variance, and the Kolmogorov-Smirnov test was used to confirm normal distribution of the data. To assess the effects of the type of instruction on dart velocity, accuracy, and maximal joint movements, a 2 (test occasion: random vs. blocked, repeated) × 4 (instruction: *V*_0_, *V*_A_, *A*_V_, *A*_0_, repeated measures) analysis of variance (ANOVA) was used. When the instruction type was found to have a significant effect, a one-way ANOVA for each test occasion was also performed. In addition, to assess differences in the timing of peak angular velocity of the three different joints a 3 × 2 × 4 ANOVA, with repeated measures, was performed. A *post hoc* test using Holm-Bonferroni probability adjustments was used to locate significant differences. In the case that the sphericity assumption was violated, the Greenhouse-Geisser adjustments of the *p* are reported in the results. All variables are expressed as mean ± SD. Statistical analysis was performed using SPSS 23.0 for Windows (SPSS, Inc., Chicago, IL).

## Results

**Figure 1:**
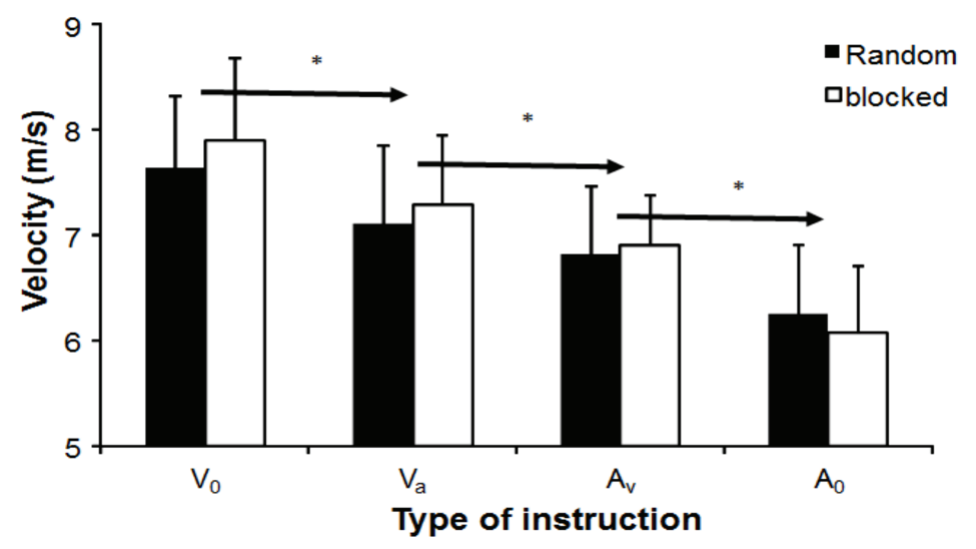
Mean (± S.D.) of dart velocity at release, which was averaged per instruction condition and testing order. * indicates a significant difference in velocity between this instruction for both testing orders with all right of the arrow sign.

The instruction significantly influenced dart velocity (*F*_3,30_ = 45.7, *p* < 0.001, *η*^2^ = 0.07). No significant effect of testing order was observed (*F*_1,10_ = 0.8, *p* = 0.39, *η*^2^ = 0.07), but a significant interaction effect was found (*F*_3,33_ = 3.3, *p* = 0.031, *η*^2^ = 0.23, Figure 1). *Post hoc* comparison revealed that, with each instruction, dart velocity decreased in both testing orders when accuracy had a higher priority (*V*_0_ → *V*_A_ → *A*_V_ → *A*_0_).

**Figure 2:**
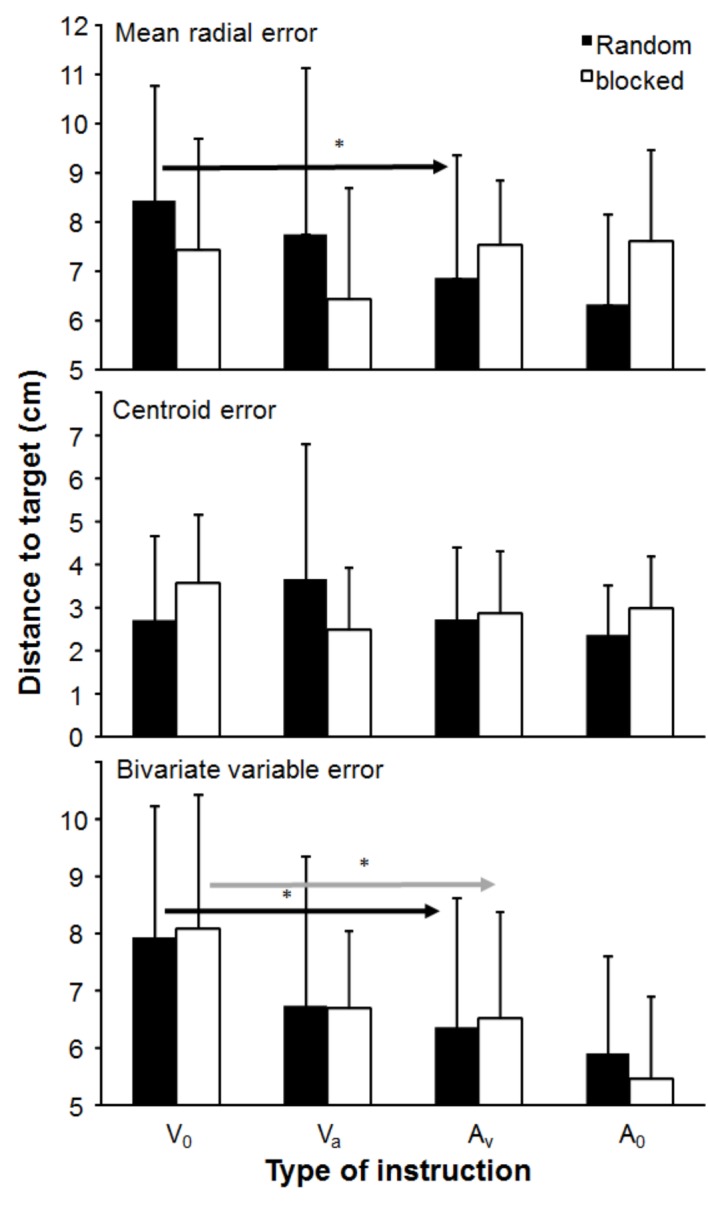
Mean (± S.D.) of accuracy expressed as mean radial error (MRE), centroid error (CE), and bivariate variable error (BVE), averaged per instruction and testing order. * indicates a significant difference in accuracy between this instruction for this testing order with all right of the arrow sign.

A significant effect of instruction (*V*_0_, *V*_A_, *A*_V_, and *A*_0_) was found only for bivariate variable error (*F*_3,30_ = 12.7, *p* < 0.0001, *η*^2^ = 0.56), a trend (0.05 < *p* < 0.10) for mean radial error (*F*_3,30_ = 2.54, *p* = 0.073, *η*^2^ = 0.19), while no significant effect was found and centroid error (*F*_3,30_ = 0.55, *p* = 0.65, *η*^2^ = 0.05, Figure 2). Furthermore, no significant effect of testing order (blocked vs. random) for any of the accuracy parameters (*F*_1,10_ ≤ 0.063, *p* ≥ 0.81, *η*^2^ ≥ 0.003) and interaction (*p* ≥ 0.073, *η*^2^ ≥ 0.19, Figure 2) was found. *Post hoc* comparison showed that bivariate radial error for both testing orders was significantly higher when the only priority was velocity (*V*_0_) compared to the instruction in which the main priority was accuracy (*A*_V_ and *A*_0_). This was also found in the random testing order for the mean radial error (Figure 2).

**Figure 3:**
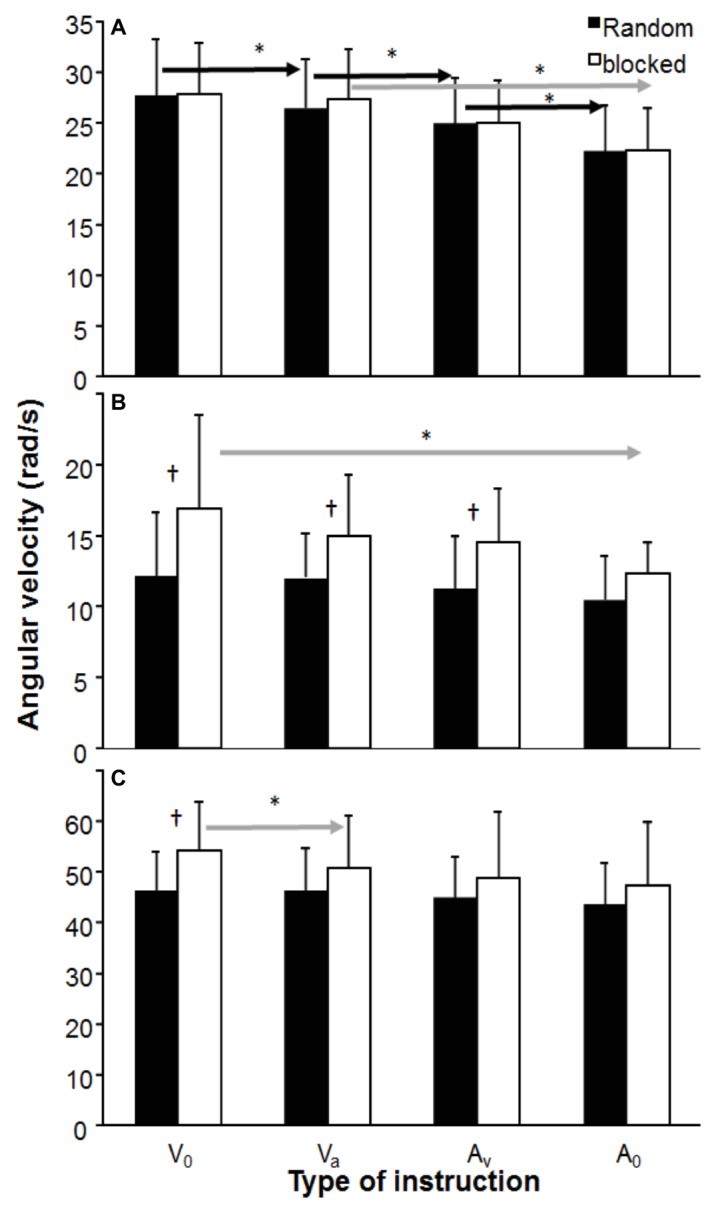
Mean (± S.D.) of **(A)** peak elbow extension, **(B)** peak wrist flexion, and **(C)** finger extension angular velocity, averaged per instruction condition and testing order. * indicates a significant difference between this instruction for this testing order with all right of the arrow sign. † indicates a significant difference between the two testing orders for this instruction.

All three peak joint movements were affected by instructions (*F*_3,33_ = 4.83, *p* ≤ 0.0025, *η*^2^ ≥ 0.31). In addition, there was a significant effect of testing order on the wrist flexion (*F*_1,11_ = 10.3, *p* < 0.001, *η*^2^ = 0.48). *Post hoc* comparison showed that in the random test order, the peak elbow extension velocity decreased with each instruction in which accuracy was of more importance in both testing orders (*V*_0_ → *V*_A_ → *A*_V_ → *A*_0_), while in the blocked testing order, a significant decrease in velocity was found only between instruction *V*_A_ and *A*_0_. In the blocked testing order, wrist flexion and finger extension were significantly higher in instruction *V*_0_ compared to instruction *A*_0_ (wrist flexion) and all other instructions (finger extension, Figure 3). Furthermore, the velocity was higher in the wrist flexion in almost all instructions except instruction *A*_0_ and the finger extension for instruction *V*_0_ (Figure 3).

**Figure 4:**
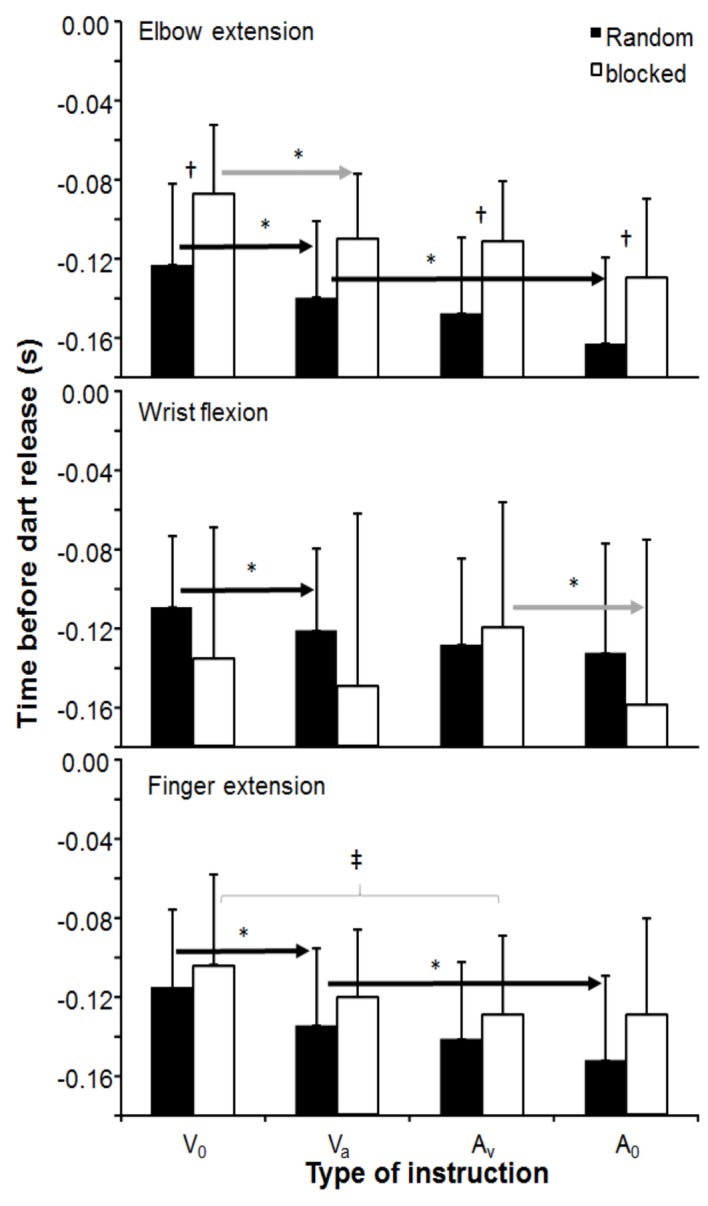
Mean (± S.D.) time of occurrence of peak elbow extension, peak wrist flexion and finger extension angular velocity, averaged per instruction condition and testing order. * indicates a significant difference between this instruction for this testing order with all right of the arrow sign. ^†^ indicates a significant difference between the two testing orders for this instruction. ^‡^ indicates a significant difference between these two instructions for this testing order.

Timing of peak angular velocity of all three joint movements was significantly affected by instruction (*F*_3,33_ ≥ 3.4, *p* ≤ 0.028, *η*^2^ ≥ 0.24), but not by joint and order. However, significant interaction effects were found for order-joint (*p* = 0.001) and order-instruction (*p* = 0.018). *Post hoc* comparison showed that time of peak elbow extension velocity in the blocked testing order occurred closer to dart release than in the random testing order (*F*_3,33_ = 6.6, *p* = 0.026, *η*^2^ = 0.37). In the random test order, timing of the peak velocities of all three joint movements in instruction *V*_0_ occurred closer to dart release than in the other instructions. Furthermore, the peak elbow and finger joint movements in instruction *V*_A_ occurred closer to dart release than in instruction *A*_0_. Also, in the blocked testing order, the time of peak elbow and finger joint movement occurred closer to dart release in instruction *V*_0_ than all other instructions (elbow) and instruction *A*_V_ (finger). Wrist flexion occurred in this testing order closer to dart release in instruction *A*_V_ compared with instruction *A*_0_ (Figure 4).

## Discussion

In this study, the effects of instructions and testing order on dart throwing performance (dart velocity and accuracy) were examined. As expected, dart velocity decreased when the priority for accuracy increased (Figure 1). However, accuracy as measured by consistency (BVE) only, increased when accuracy was the main priority (Figure 2). Testing order seemed to influence peak joint kinematics of wrist flexion, elbow and finger extension (Figure 3), and time of occurrence of the elbow extension (Figure 4).

Throwing velocity was affected by instruction as expected, when emphasis was placed on accuracy, the velocity was reduced (Figure 1), and thereby followed Fitts’ law (Fitts, 1954). The changes in velocities between instructions was similar, indicating that in dart throwing, a clear distinction in throwing velocity is possible when the set priority, based on instructions, is different. These results differed from studies conducted by van den Tillaar and Ettema on overarm throwing in handball players, which showed that instructions emphasizing both accuracy and velocity led to a similar performance (van den Tillaar and Ettema, 2003a,b, 2006). The different outcomes could be explained by the type of movement required dart and handball throwing. In the present study, only the upper extremity could be used, while in handball throwing, the entire upper body is used, which also involves rotations in the pelvis and trunk. Handball throwing involves more joint segments and muscles, thereby increasing the degrees of freedom that have to be controlled (van den Tillaar and Ettema, 2007), possibly making it more difficult for the subject to distinguish between instructions that have only subtle distinctions (emphasizing velocity first and secondly accuracy or the opposite).

According to Fitts’ law (Fitts, 1954), we are limited in processing information and execution of movements. In dart throwing, the movement is not very complex as in overarm throwing in handball. This law also says that accuracy would increase when accuracy is the main priority. However, this was only partly shown in this study. Consistency (bivariate variable error) increased when accuracy was the main priority, while the bias (centroid error) was the same, and no significant difference in mean radial error were found (Figure 2). However, a closer examination of the mean radial error revealed that the error decreased under the random order condition when accuracy was prioritized, while no significant difference was observed in the blocked condition. This difference of development was shown by a nonsignificant interaction effect between the two conditions (*p* = 0.073). These differences can be explained by the idea that in the blocked condition, subjects could adjust their movements based upon the previous throw. By adjusting their movements, the subjects could overcompensate (Binsch et al., 2009) and throw above the target when the previous throw hit under the target. This phenomenon of overcompensation is normal in aiming tasks (Binsch et al., 2009; Toner et al., 2013) in which subjects throw too high because they want to avoid throwing too low again. In the present study, this was shown by insignificant changes in the mean radial error between the instructions in the blocked testing protocol.

In throwing velocity, the effect of the instructions between blocked and random order was evident by the significant interaction (Figure 1). It seems that in the blocked condition, the subjects tended to throw faster in the first three instructions when velocity was the priority, while in the last instruction, when accuracy was the only priority, the subjects threw slower than in the random order condition. This indicates that the subjects used other movement strategies, which were clearly discernable in changes in maximal angular velocities of the joint movements and their occurrence before dart release.

Different movement strategies were evident by changes in peak angular velocity of the three joint movements and their occurrence. In general, all maximal velocities decreased when accuracy was the priority (Figure 3), indicating that these joint movement changes caused the observed decrease in dart throwing. However, when closely examining the peak velocities of the wrist extension and finger extension, the peak velocities in the blocked order condition decreased when accuracy was prioritized, while no changes occurred in the random order condition (Figure 3). Furthermore, the peak angular velocity of the wrist flexion and finger extension was higher in the instructions that prioritized velocity in the blocked condition compared with the random condition. In addition, timing of peak elbow extension velocity was closer before dart release in the blocked condition, which could have a positive effect on dart throwing. This indicates that in the blocked order, it is clearer that movement adjustments occur that favor the priority set by the instruction than in the random order. Lee and Magill (Lee and Magill, 1983, 1985) suggested that the trial-to-trial repetition of a task in blocked order reduces the likelihood that information specific to that task is forgotten at the beginning of each subsequent trial. Because of that, little reconstructive activity in terms of movement planning is necessary because the requisite information already resides in working memory. Thus, little interaction between working memory and long-term memory is warranted during blocked practice (Li and Wright, 2000).

These changes in peak velocity between the different joints caused an order-joint and joint-instruction interaction as shown by changes that occurred in each testing order. In the blocked order, in the peak extension of the elbow occurred closer to dart release than the wrist flexion, while in the random order, a longer peak elbow extension occurred before dart release compared to the other two joint movements (Figure 4). All these differences indicate that it is important to specify how attempts were organized random or in a blocked order since it has an obvious effect upon movement strategies and thereby an effect upon the outcome parameters (velocity and accuracy). It seems that by low contextual interference (blocked condition) during testing, it is easier to make adjustments of maximal angular velocities in the distal joint movements (wrist and finger), while in high contextual interference (random condition), only adaptations in the proximal joint (elbow) occur (Figure 3). This low-high contextual interference (Shea and Morgan, 1979) also influences timing of occurrence of peak velocity of the involved joints differently. In most aiming overarm actions, a proximal-to-distal sequence occurs in the joint movements to transfer energy from the proximal to distal segments to achieve the highest possible velocity (Jöris et al., 1985; Fradet et al., 2004; van den Tillaar and Ettema, 2009b). In dart throwing, it seems that the occurrence of peak elbow and finger extension and wrist flexion occur at approximately the same time (Figure 4). However, in blocked condition, when the only priority is velocity (*V*_0_), the peak elbow extension occurred significantly closer to dart release than wrist flexion; whereas, in the random condition peak, wrist flexion occurs significantly closer to dart release than elbow and finger extension when the only priority was accuracy. The peak elbow extension occurring closer to dart release than the other two movements in the blocked condition in instruction *V*_0_ is explainable by the fact that when accuracy is of no importance throwing velocity is easier to generate by a long lever, propelled by large muscle groups. The elbow joint has a much longer lever than finger and wrist joints and is controlled by large muscle groups (e.g., triceps and biceps), and thereby a natural choice to use more when focusing on velocity. This is in line with studies on overarm throwing in which internal rotation, elbow extension, and trunk rotation contribute mostly to the maximal throwing velocity (van den Tillaar and Ettema, 2007; Wagner et al., 2010). In the random condition, the peak wrist flexion occurring closer to dart release than the other two joint movements during instruction *A*_0_ can be explained by the fact that timing of peak wrist flexion did not change when accuracy was involved in the instruction (*V*_A_ − *A*_0_), while the occurrence of the peak elbow and finger extension changed (Figure 4). This changed the timing sequence between the three joints. The timing of occurrence of peak wrist flexion did not change much when accuracy was involved indicating that this joint movement is very important for the accuracy of the dart throw. This indicates that both instruction and testing order had an effect on the dart throwing strategy.

The level of expertise in dart throwing of the subjects may explain why, when accuracy was prioritized, only an effect for increased consistency was observed, not for the mean radial error, thereby not fully following Fitts’ law. They were not elite dart throwers, but they had some dart throwing experience, which influenced the accuracy performance. On average, they missed the target by 3 cm (CE) and a standard deviation of 7 cm around bulls eye (MRE), which is probably much higher compared to elite dart throwers. Most likely, this also influences the kinematics of the throws. The participants in the present study possibly have a higher variability in movement kinematics compared to highly skilled dart players and thereby so much variability in execution of the different throws that the mean radial error did not show a clear speed-accuracy trade-off. This was in accordance with Toner et al. (2013) who showed that over compensatory behavior was more prevalent amongst low-skilled than high-skilled golfers and thereby could very much influence throwing accuracy, especially in the blocked condition. This is a clear limitation of the present study. Furthermore, in the present study, no counter balanced crossover design was used in which half of the subjects started with one condition, while the others started with the other condition. Testing all subjects at the random condition, the first day could lead to a learning effect for the second day of testing (Shea and Morgan, 1979; Ollis et al., 2005), which is another limitation of the study. However, only a few throws in each condition were performed and there were at least 2 weeks between the two testing occasions, which would minimize the eventual learning effect. Furthermore, other studies have found that the learning effect after such a period and amount of testing are minimal or nonexistent (Lee and Simon, 2004; Maslovat et al., 2004; Russell and Newell, 2007).

In a future study, elite dart throwers must be included and testing should be performed in a counter balanced cross over design, to investigate if they show the same relationships as in our study or if they show other relationships, since they are trained to throw accurately, not as hard as possible.

## Conclusion

In conclusion, instructions that emphasized velocity and/or accuracy showed a clear speed-accuracy trade-off in dart throwing and thereby followed the Fitts’ law (Fitts, 1954). Testing order had a slight effect on the speed-accuracy trade-off. The blocked testing order seemed to increase performance outcomes that favored the priority set by the instruction than in the random test order. These differences are based upon adjustments of joint movements, which are based upon the knowledge of the previous attempts. These adjustments are evident between the different instructions, by changes in execution of peak wrist flexion, elbow, and finger extension timing.

## Ethics Statement

This study was carried out in accordance with the recommendations of “name of guidelines, name of committee” with written informed consent from all subjects. All subjects gave written informed consent in accordance with the Declaration of Helsinki. The protocol was approved by the Norwegian Centre for Research Data.

## Author Contributions

RT contributed to idea, data collection, analysis, and writing the manuscript, while TA contributed with the idea, data collection and discussing the results and writing the manuscript.

### Conflict of Interest Statement

The authors declare that the research was conducted in the absence of any commercial or financial relationships that could be construed as a potential conflict of interest.
